# Loss of Mitochondrial Tumor Suppressor Genes Expression Is Associated with Unfavorable Clinical Outcome in Head and Neck Squamous Cell Carcinoma: Data from Retrospective Study

**DOI:** 10.1371/journal.pone.0146948

**Published:** 2016-01-19

**Authors:** Ishrat Mahjabeen, Mahmood Akhtar Kayani

**Affiliations:** Cancer Genetics & Epigenetics Research Group, Department of Biosciences, COMSATS Institute of Information Technology, Islamabad, Pakistan; Ludwig-Maximilians University, GERMANY

## Abstract

Mitochondrial genes play important roles in cellular energy metabolism, free radical generation, and apoptosis. Dysregulation of these genes have long been suspected to contribute to the generation of reactive oxygen species (ROS), increased proliferation and progression of cancer. A family of orthologues of yeast silent information regulator 3 (SIRT3), 4 (SIRT4) and mitochondrial tumor suppressor 1 (MTUS1) are important mitochondrial tumor suppressor genes which play an important role in the progression of multiple cancers. However, their role in the development of oxidative stress, enhanced proliferation and progression of head and neck squamous cell carcinoma (HNSCC) has not yet been studied. In this study we aimed to test the association between reduced mitochondrial tumor suppressor genes’ activities and enhancement in tissue oxidative stress and cell proliferation in HNSCC cases. The expression of mitochondrial tumor suppressor genes (SIRT3, SIRT4 and MTUS1), mitochondrial DNA repair gene (OGG1-2a) and a proliferation marker (Ki-67) was studied in a study cohort of 120 HNSCC patients and controls with reverse transcriptase polymerase chain reaction (RT-PCR) and real-time PCR (qPCR) in order to determine the potential prognostic significance of these genes. A statistically significant downregulation of SIRT3 (p<0.001), SIRT4 (p<0.0001), MTUS1 (p<0.002) and OGG1 (p<0.0001) was observed in HNSCC compared to control samples. Ki-67 was also overexpressed (p<0.0001) in HNSCC versus control samples. Additionally, to explore gene–gene relationship, we observed a positive spearmen correlation between SIRT3 versus SIRT4 (r = 0.523***, p<0.0001), SIRT3 versus MTUS1 (r = 0.273***, p<0.001), SIRT3 versus OGG1-2a (r = 0.213*, p<0.03), SIRT4 versus OGG1 (r = 0.338***, p<0.0001) and MTUS1 versus OGG1-2a (r = 0.215*, p<0.03) in HNSCC cases. A negative spearman correlation was observed between OGG1 versus Ki-67 (r = -0.224**, p<0.01) and OGG1-2a versus Ki-67 (r = -0.224**, p<0.01) in HNSCC cases. Here we report that the deregulation of mitochondrial tumor suppressor genes (SIRT3, SIRT4 and MTUS1) in relation to decreased expression of mitochondrial DNA repair gene OGG1-2a and increased proliferation (measured by proliferation marker Ki-67) may be considered important factors in the development of head and neck squamous cell carcinoma.

## Introduction

Mitochondrial dysfunction has long been linked to cancer and a number of mechanisms have been reported to be involved in this process [1]. Among these mechanisms, dysregulation of different mitochondrial tumor suppressor (TS) genes have been suggested. Sirtuins, a family of orthologues of yeast silent information regulator 3 (Sirt3) and 4 (Sirt4) are important TS genes located in mitochondria [2–4]. SIRT3 and SIRT4 are tumor suppressor genes and the primary mitochondrial deacetylase which regulates metabolic function through variety of mechanisms [5]. SIRT3 directs post-translational modifications via protein deacetylation [6], Sirt4 affects its targets largely through NAD-dependent ADPribosylation [5]. It has been proposed that mitochondrial proteins, such as SIRT3 and SIRT4, may function as critical regulators at the crossroads between metabolism, aging, and cancer [7]. Significant decrease in SIRT3 levels has been observed in different human tumors such as breast tumor, hepatic tumor and lung tumors compared to their respective normal tissue controls [8–10]. Same is the case with SIRT4 gene, where SIRT4 mRNA expression has been observed reduced in several malignancies, including breast, colon, bladder, gastric, ovarian, lung and thyroid cancers [11, 12]. However, their role in the progression of HNSCC has not yet been studied.

In addition to these, mitochondrial tumor suppressor 1 (MTUS1) is another important TS gene and is localized at 8p22, a chromosomal region frequently deleted in tumors [13]. MTUS1 encodes a family of angiotensin II (AT2) receptor-interacting proteins (ATIP). Alternative exon utilization of this gene leads to 5 known transcript variants that code for 5 different protein isoforms of ATIP (ATIP1, ATIP2, ATIP3a, ATIP3b and ATIP4). [14, 15]. MTUS1 gene is a candidate mitochondrial gene which controls important process such as proliferation, invasion and progression [16, 17]. Previous studies have shown that MTUS1 expression levels are reduced in cancers from colon [18, 19] prostate [20], breast cancer [21] and tongue [16]. However, the implications of mitochondrial tumor suppressor genes SIRT3, SIRT4 and MTUS1 in head and neck cancer are largely unknown.

The present study was designed to study the expression pattern of selected mitochondrial tumor suppressor genes in head and neck cancer and to investigate the association of SIRT 3, SIRT4 and MTUS1 gene expression with the clinical characteristics and prognosis of head cancer patients. Furthermore, expression levels of selected mitochondrial tumor suppressor genes were correlated with generation of oxidative stress (measured by OGG1-2a level) and with proliferation process (measured by Ki-67 level) in head and neck cancer patients to illuminate the association between the mitochondrial genes alterations, ROS generation and proliferation pathway in head and neck tumorigenesis.

## Material and Methods

### Tumor sample collection

Tumors were collected from 120 head and neck cancer patients, after surgery from Pakistan Institute of Medical Sciences (PIMS) Islamabad. Samples of tumor core, the invasive edge of tumor and microscopically healthy mucosa (control) were obtained from each surgical section and stored in RNA at -80°C. Presence of tumor cells in the collected tissues was rectified by examination of frozen sections following hematoxylin and eosin stain (HE stain) by a consultant pathologist (S1 Fig). Whereas samples of control were obtained from macroscopically confirmed uninvolved healthy area more than 2cm away from the tumor (S1 Fig). Specifically designed performa was filled for each patient and proper consent was obtained. Information about gender, age and smoking status was also recorded. Clinical characterization of the patients is summarized in Table 1.

**Table 1:** Demographic and clinical characterization of the cohorts.

Variables	Cancer Patients N (%)	Healthy Controls N (%)
**Age**		
Mean	52±0.5	52±0.5
<40 n (%)	28 (23)	29 (24)
>40 n (%)	92 (77)	91 (76)
**Gender**		
Male n (%)	72 (60)	74 (62)
Female n (%)	48 (40)	46 (38)
**Area of Cancer**		
Oral cavity n (%)	65 (54)	
Pharynx n (%)	21 (18)	
Larynx n (%)	34 (28)	
**Clinical Staging**		
I-II n (%)	49 (41)	
III-IV n (%)	71 (59)	
**TNM Staging**		
**T Staging**		
T1-T2 n (%)	48 (40)	
T3-T4 n (%)	72 (60)	
**N Staging**		
N0 n (%)	49 (41)	
N1-N2 n (%)	71 (59)	
**M Staging**		
M0 n (%)	88 (73)	
M1-M2 n (%)	33 (27)	
**Survival Data**		
Cured n (%)	41 (34)	
Under treatment n (%)	46 (38)	
Deceased n (%)	33 (28)	
**Grades**		
Poorly-differentiated n (%)	32 (27)	
Moderate n (%)	41 (34)	
Well-differentiated n (%)	47 (39)	

### Ethical Standard

The study was conducted with a prior approval from the institutional ethical review board of COMSTAS Institute of Information Technology (CIIT) Islamabad. Members of this committee include Dean ORIC (Office of Research Innovation and Commercialization) Prof. Dr. Raheel Qamar (convener), Prof. Dr. Mahmood A Kayani (Chairman Deptt of Biosciences), Dr. Faheem Tahir (Deputy Director, NIH) and Dr. Tayyaba Yasmin (Associate Head of the Department. All the samples were collected after a signed, informed consent from all participants of the study. The ethical review board approved the consent procedure & execution of project on head and neck cancer.

### RNA extraction and cDNA synthesis

RNA isolation was carried out from tumor samples of HNSCC and controls using standard Trizol reagent method [22]. Extracted RNA was analyzed on 1% TAE gel to confirm the isolation of RNA. After isolation, yield of RNA was quantified with the help of UV spectrophotometer. Absorbance was measured at wavelengths of 260 and 280nm. Extracted RNA was stored at -80°C. cDNA was synthesized by SuperScript III First Strand Synthesis system (Invitrogen). Single stranded cDNA was synthesized from purified total RNA and stored at 4°C.

### Reverse Transcriptase Polymerase Chain Reaction (RT-PCR)

Reverse transcriptase polymerase chain reaction was used to analyze RNA expression. In RT-PCR, the expression of gene is quantified by the synthesis of cDNA. RT-PCR was carried out to assess expressional variations of SIRT3, SIRT4, MTUS1, OGG1-2a and Ki-67 gene in head and neck tumor samples. Primers specific for gene SIRT3, SIRT4, MTUS1, OGG1-2a gene, proliferation marker Ki-67 and for reference gene β-actin (internal control) were obtained from IDT DNA Technology and optimized for specific RT-PCR reaction (S1 Table). The reaction conditions optimized for this reaction were; initial denaturation for 2 minutes at 95°C and 35 cycles of 94°C for 15 seconds, annealing at 56°C for 15 seconds, 72°C for 30 seconds and final extension of at 72°C for 10 minutes. Amplification of internal control β-actin was also carried out to check the specificity of the reaction. Amplified products were run on 2% agarose gel electrophoresis after the completion of PCR reaction. Their densities were analyzed using the Quantity One software (Bio-Rad Laboratories, Inc., Hercules, CA, USA).

### Quantitative polymerase chain reaction (q-PCR)

For quantitative PCR, primers specific for gene SIRT3, SIRT4, MTUS1, OGG1-2a gene, proliferation marker Ki-67 and for reference gene β-actin (internal control) were obtained from IDT DNA Technology and optimized for specific q-PCR (S1 Table). Each qPCR was performed in a 10μL reaction mixture containing 1μL of cDNA, 1μL of each forward and reverse primer, 5μL of Syber green master mix, 2μL of RNase free water. qPCR was performed using Step 1 plus PCR system (Applied biosystem) at 56°C with standard conditions and quality of each qPCR reaction was checked by melt curve analysis (S2 Fig). Relative mRNA expression of SIRT3, SIRT4, MTUS1, OGG1-2a gene and Ki-67 gene was computed using 2^-∆∆Ct^ analysis method where β-actin was used as reference gene [23].

### Statistical analysis

χ2-test, one-way analysis of variance and Tukey s’ post hoc test was used to assess the association of SIRT3, SIRT4, MTUS1, OGG1-2a and Ki-67 expression with clinical and histopathological parameters (e.g.TNM and grade). Spearman correlation coefficient was used to assess correlations among the gene expression and clinical and histopathological parameters. Statistical analysis was performed using GraphPad Prism5 software and SPSS 6.0 software package.

## Results

**Fig 1:**
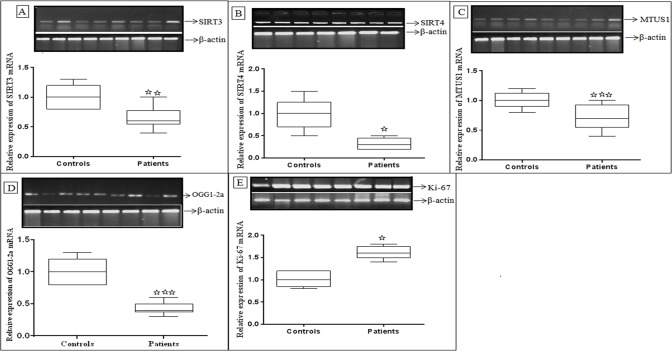
Reverse transcriptase PCR reaction of (A) SIRT3, (B) SIRT4, (C) MTUS1, (D) OGG1-2a and (E) Ki-67 in HNSCC cases compared with adjacent control tissues.β-actin was used as internal controls. * p < 0.05, ** p < 0.01 and *** p < 0.001. The p values were computed using one way ANOVA and χ2-test.

Using semi quantitative reverse transcriptase PCR (RT PCR), gene expression levels of three mitochondrial tumor suppressor genes such as SIRT3, SIRT4 and MTUS1 were determined in 120 HNSCC cases and control tissues. A significant down-regulated expression of SIRT3 (p<0.007), SIRT4 (p<0.004) and MTUS1 (p<0.0009) was observed in HNSCC cases compared to adjacent uninvolved non-cancerous control tissue samples. In addition to this relative expression of selected mitochondrial DNA repairing gene OGG1-2a and proliferation markers Ki-67 were also determined in same study cohort and significant down-regulated expression of OGG1-2a (p<0.0001) was observed in HNSCC tumors compared to control tissue samples. In case of proliferation marker *Ki-67*, a significant up-regulated (p<0.01) expression was observed in tumor tissues compared to adjacent uninvolved non-cancerous control tissue samples (Fig 1).

**Fig 2:**
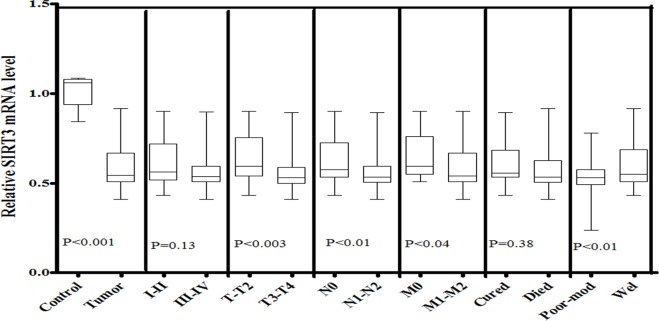
mRNA expression of SIRT3 in HNSCC cases of study cohort. Box plot comparing the SIRT3 mRNA levels of HNSCC and normal control samples, HNSCC samples with clinical stage I–II and clinical stage III–IV, with different T stages, with lymph node (N1–N2) and without lymph node (N0), with metastasis (M1-M2) and without metastasis (M0), with survival status and HNSCC samples with different grades. The p-values were computed using one-way analysis of variance and Tukey s’ post hoc test.

In order to further confirm the expression patterns of SIRT3, SIRT4, MTUS1, OGG1-2a and Ki-67 gene in same study cohort, quantitative PCR (qPCR) was also performed. A significant down-regulated (p<0.001) expression of SIRT3 was observed in HNSCC tissues compared to control tissue samples. Statistical significant decrease in SIRT3 expression was observed in relation to T-stage (p<0.003), N-stage (p<0.01) and M-stage (p<0.04) of the head and neck tumors. SIRT3 expression was also observed significantly lower in poor-moderately differentiated (p<0.01) tumors than in well-differentiated tumor of head and neck region as shown in Fig 2.

**Fig 3:**
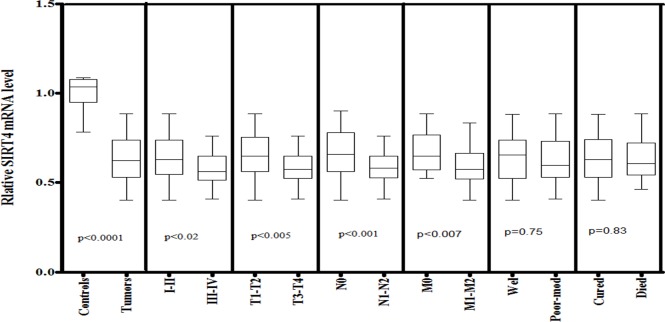
mRNA expression of SIRT4 in HNSCC cases of study cohort. Box plot comparing the SIRT4 mRNA levels of HNSCC and normal control samples, HNSCC samples with clinical stage I–II and clinical stage III–IV, with different T stages, with lymph node (N1–N2) and without lymph node (N0), with metastasis (M1-M2) and without metastasis (M0), with survival status and HNSCC samples with different grades. The p-values were computed using one-way analysis of variance and Tukey s’ post hoc test.

The SIRT4 expression was observed significantly (p<0.0001) lower in HNSCC tissues when compared to normal tissue samples. The expression level of SIRT4 was significantly (p<0.02) lower in late-stage (III–IV) than in early-stage tumors (I–II). A similar decrease in SIRT4 expression was also observed in larger tumor (T3–T4, p<0.005) as compared to smaller tumors (T1–T2). Furthermore, statistical significant decrease in SIRT4 mRNA level was also observed in the tissues with positive for lymph node involvement (N1-N2, p<0.001) and with positive for metastasis (M1-M2, p<0.007) compared to those with negative for lymph node involvement (N0) and metastasis (M0) respectively (Fig 3).

**Fig 4:**
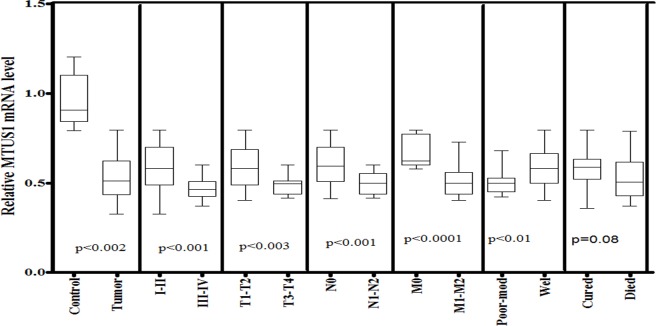
mRNA expression of MTUS1 in HNSCC cases of study cohort. Box plot comparing the MTUS1 mRNA levels of HNSCC and normal control samples, HNSCC samples with clinical stage I–II and clinical stage III–IV, with different T stages, with lymph node (N1–N2) and without lymph node (N0), with metastasis (M1-M2) and without metastasis (M0), with survival status and HNSCC samples with different grades. The p-values were computed using one-way analysis of variance and Tukey s’ post hoc test.

MTUS1 gene was observed significantly down-regulated (p<0.002) in HNSCC tissues as compared to normal tissue samples. The expression level of MTUS1 gene was significantly lower in late stage (III-IV) than in early stage disease (I-II). Similar decrease in MTUS1 expression was also observed in large (T3-T4, p<0.003) tumors as compared to smaller (T1-T2) tumors. In case of lymph node and metastatic status, a significantly lower MTUS1 level was observed in patients with positive for lymph node involvement (N1-N2, p<0.001) and with positive for metastasis (M1-M2, p<0.0001) as compared to patients with negative lymph node (N0) involvement and with negative metastasis (M0) respectively. Furthermore, a significant lower level of MTUS1 was also observed in case of advance tumor grade (poor-moderately differentiated tumors, p<0.01) as compared to well differentiated tumors as shown in Fig 4.

**Fig 5:**
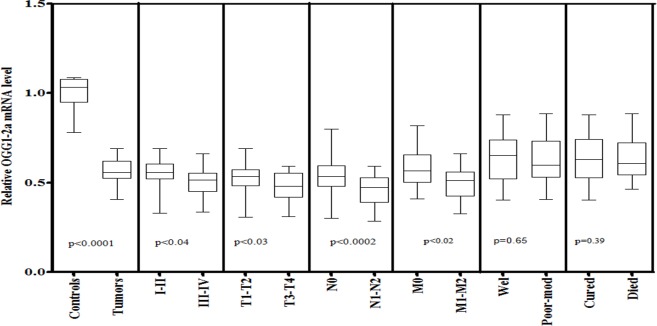
mRNA expression of OGG1-2a in HNSCC cases of study cohort. Box plot comparing the OGG1-2a mRNA levels of HNSCC and normal control samples, HNSCC samples with clinical stage I–II and clinical stage III–IV, with different T stages, with lymph node (N1–N2) and without lymph node (N0), with metastasis (M1-M2) and without metastasis (M0), with survival status and HNSCC samples with different grades. The p-values were computed using one-way analysis of variance and Tukey s’ post hoc test.

In case of mitochondrial DNA repair gene OGG1-2a, significantly lower level (p<0.0001) of OGG1-2a was observed in HNSCC tissues as compared to control tissue. The expression level of OGG1-2a was significantly lower in advance clinical stage (III-IV, p<0.04) and tumor stage (T3-T4, p<0.03) as compared to early clinical stage (I-II) and tumor stage (T1-T2) respectively. Furthermore, significantly lower mRNA level of OGG1-2a was observed in patients with positive lymph node status (N1-N2, p<0.0002) and with positive metastasis stage (M1-M2, p<0.02) compared to patients with negative lymph node status (N0) and with negative metastasis stage (M0) as shown in Fig 5.

**Fig 6:**
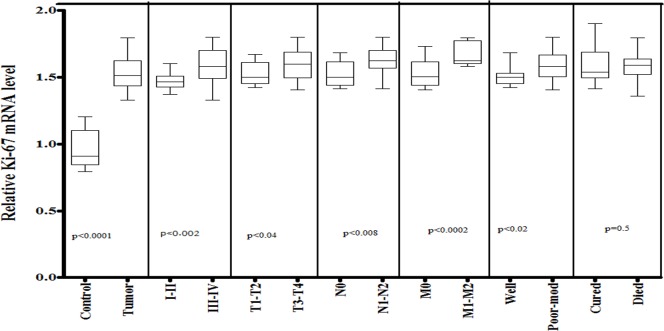
mRNA expression of Ki-67 in HNSCC cases of study cohort. Box plot comparing the Ki-67 mRNA levels of HNSCC and normal control samples, HNSCC samples with clinical stage I–II and clinical stage III–IV, with different T stages, with lymph node (N1–N2) and without lymph node (N0), with metastasis (M1-M2) and without metastasis (M0), with survival status and HNSCC samples with different grades. The p-values were computed using one-way analysis of variance and Tukey s’ post hoc test.

Moreover, expression level of proliferation marker Ki-67 was also observed in 120 HNSCC tissue samples and adjacent non-cancerous tissues as controls. A significant up-regulated (p<0.0001) expression of Ki-67 was observed in HNSCC tissue samples compared to control samples. The expression level of Ki-67 was significantly (p<0.002) higher in late-stage (III-IV) than in early-stage (I–II). A similar increase in Ki-67 expression was also observed in larger (T3-T4, p<0.04) tumor tissues as compared to smaller (T1–T2) tumors. Statistically significant increase in Ki-67 mRNA level was observed in tissues with positive for lymph node involvement (N1-N2, p<0.008) and with positive for metastasis (M1-M2, p<0.0002) as compared to those with no lymph node involvement (N0) and with no metastasis (M0). Furthermore, significant up-regulated expression of Ki-67 was also observed in poor-moderately differentiated tumors (p<0.02) compared to well differentiated tumors. The expression levels of Ki-67 were shown in Fig 6.

To explore gene-gene interaction, we observed a positive spearmen correlation between SIRT3 versus SIRT4 (r = 0.523***, p<0.0001), SIRT3 versus MTUS1 (r = 0.273***, p<0.001), SIRT3 versus OGG1-2a (r = 0.213*, p<0.03), SIRT4 versus OGG1-2a (r = 0.338***, p<0.0001) and MTUS1 versus OGG1-2a (r = 0.215*, p<0.03) in HNSCC cases (* indicates the level of significance of spearmen correlations as calculated by SPSS). A negative spearman correlation was observed between OGG1-2a versus Ki-67 (r = -0.224**, p<0.01) and OGG1-2a versus Ki-67 (r = -0.224**, p<0.01) in HNSCC cases. No significant correlation was observed between SIRT3 versus Ki-67, SIRT4 versus MTUS1and MTUS1 versus Ki-67 in HNSCC cases.

In case of gene–clinicopathological characteristic relationship, significant negative correlation was observed between SIRT3 versus N stage (r = -0.226**, p<0.01), SIRT4 versus N stage (r = -0.261***, p<0.001), MTUS1 versus N stage (r = -0.214*, p<0.05) and OGG1-2a versus N stage (-r = 0.378***, p<0.001) in HNSCC cases. Furthermore, significant negative correlation was also observed between SIRT3 versus M stage (-0.30***, p<0.001), SIRT4 versus M stage (-0.185*, p<0.04), MTUS1 versus M stage (-0.289**, p<0.001), OGG1-2a versus M stage (0.216*, p<0.03) and Ki-67 versus M stage (0.24**, p<0.008) in HNSCC cases as shown in Table 2.

**Table 2:** Correlations between mitochondrial tumor suppressor genes (*SIRT3*, *SIRT4*, *MTUS1*), mitochondrial DNA repair gene (*OGG1-2a*), proliferation marker (*Ki-67*) expression and clinicopathological characteristics of primary HNSCC^†^.

T stage	C stage	N stage	M stage	Grade	Survival	*SIRT3*	*SIRT4*	*MTUS1*	*OGG1-2a*	*Ki-67*
**T stage**	0.665***	0.501***	0.078	0.222*	-0.006	0.11	-0.074	0.127	0.005	0.059
**C stage**		0.387***	0.053	0.204*	0.021	0.138	-0.1	0.085	0.048	0.053
**N stage**			0.380***	0.037	-0.051	-0.226**	-0.261***	-0.214*	-0.378***	0.078
**M stage.**				0.125	0.01	-0.30***	-0.185*	-0.289***	-0.216*	0.24**
**Grade**					0.154	0.007	**-**0.15	0.105	0.012	0.094
**Survival**						0.15	0.05	0.08	-0.083	0.100
***SIRT3***							0.523***	0.273***	0.213*	**-**0.054
***SIRT4***								0.08	0.338***	-0.108
***MTUS1***									0.125*	-0.674***
***OGG1-2a***										0.224*
***Ki-67***										

**†** Spearman Correlation Coefficients. The expression levels of *SIRT3*, *SIRT4*, *MTUS1*, *OGG1-2a* and *Ki-67* for Patient Cohort were based on the relative mRNA level.

* p < 0.05

** p < 0.01 and

*** p < 0.001. The p values were computed using one way ANOVA and χ2-test.

From this correlation study, it is important to note that in HNSCC, inverse correlation was consistently observed between SIRT3, SIRT4, MTUS1 and OGG1-2a expression N stage and M stage. However, positive correlation was observed between Ki-67 and N stage and M stage as illustrated in Table 3, statistical analysis revealed that the SIRT3, SIRT4, MTUS1, OGG1-2a and Ki-67 expression levels in head and neck cancer patients is associated with lymph node status and metastasis status of HNSCC cases.

**Table 3:** Association of expression deregulation of SIRT3, SIRT4, MTUS1, OGG1-2a and Ki-67 in HNSCC and lymph node and metastasis.

Parameters		N	Average	Variance	p-value
**SIRT3**	N0 N1-N2	49 71	0.62 0.55	0.98 1.0	**<0.01**
	M0 M1-M2	88 33	0.64 0.49	0.760.97	**<0.0004**
**SIRT4**	N0 N1-N2	49 71	0.67 0.60	0.980.84	**<0.0005**
	M0 M1-M2	88 33	0.59 0.52	0.810.79	**<0.0003**
**MTUS1**	N0 N1-N2	49 71	0.61 0.50	1.050.77	**<0.0002**
	M0 M1-M2	88 33	0.66 0.52	0.790.82	**<0.0001**
**OGG1-2a**	N0 N1-N2	49 71	0.54 0.46	0.781.05	**<0.0008**
	M0 M1-M2	88 33	0.60 0.53	0.810.79	**<0.002**
**Ki-67**	N0 N1-N2	49 71	1.54 1.62	1.452.09	**<0.0001**
	M0 M1-M2	88 33	1.53 1.66	2.111.35	**<0.0004**

## Discussion

Mitochondrial genes variations have long been suspected to play an important role in the development and progression of cancer [24]. Here, we report a role for mitochondrial genes and specifically, for sirtuins family (SIRT3 and SIRT4) and MTUS1 in head and neck carcinogenesis. Although there is emerging evidence for a role of several mitochondrial genes in carcinogenesis, their role in head and neck cancer has not been reestablished. In this study, we explored the effect of expression dysregulation of mitochondrial TS genes on HNSCC risk with interaction of altered levels of mitochondrial DNA repairing gene, OGG1-2a as a measure of reactive oxygen species (ROS) exposure and tissue oxidative stress. In the first step expression levels of SIRT3, SIRT4 and MTUS1 genes were assessed by RT-PCR and qPCR. Significant down-regulated expression of mitochondrial TS genes such as SIRT3, SIRT4 and MTUS1 was observed in HNSCC samples compared to control samples. This reduced SIRT3, SIRT4 and MTUS1 expression was also observed when correlated with the other clinico-pathological parameters.

Reduced mRNA levels of mitochondrial TS genes have earlier been reported in various malignancies including breast, colon, gastric, and hepatic cancers [8, 12]. The downregulation of SIRT3 and SIRT4 may lead to enhanced acetylation status and ROS generation in mitochondria, which are tightly associated with higher cancer risk [25]. Loss of function or genetic deletion of these mitochondrial tumor suppressor genes such as SIRT3, SIRT4 and MTUS1 may result in a mismatch of mitochondrial energy metabolism, culminating in a cell phenotype permissive for transformation and tumorigenesis [12]. In present study, we also observed a significant positive correlation between the SIRT3, SIRT4 and MTUS1 mRNA expression level by spearman correlation coefficient.

In second step, we analyzed the expression level of mitochondrial DNA repair gene OGG1-2a in HNSCC cases and respective controls using RT-PCR and qPCR analysis. Significant down-regulated expression of OGG1-2a was observed in HNSCC cases when compared with respective controls. Accumulating evidence indicates that low hOGG1 expression is associated with increased 8-oxoG lesions in genomic DNA and may give rise to a mutator phenotype in which mutations will arise faster in cells with a low hOGG1 expression [26, 27]. A Previous study has shown that the reduction of hOGG1 expression is expected to decrease mtDNA repair and cellular survival in response to oxidative damage [28]. One step forward, expression levels of OGG1-2a was correlated with expression levels of mitochondrial tumor suppressor genes such as SIRT3, SIRT4 and MTUS1 gene by spearman correlation coefficient and a significant positive correlation was observed between OGG1-2a and SIRT3, SIRT4 and MTUS1 genes which shown that OGG1-2a activity is regulated by SIRT3, SIRT4 and MTUS1 genes. It has earlier been observed that SIRT3 activation has impact on the repair of mtDNA through its ability to deacetylate OGG1, a DNA glycosylase important in BER. Loss of SIRT3 results in increases of acetylation and degradation of OGG1 [29] which ultimately results in increased ROS generation and carcinogenesis.

It is known that loss of mitochondrial TS gene levels resulted in increased oxidative stress, tumor cell proliferation and tumorigenesis [30, 31]. In order to confirm this hypothesis, we analyzed the expression pattern of proliferation marker Ki-67 in the same study cohort and correlated this expression pattern with mitochondrial tumor suppressor genes (SIRT3, SIRT4, MTUS1) and mitochondrial DNA repairing gene OGG1-2a. Significant up-regulated expression of ki-67 was observed in HNSCC cases when compared with respective controls and this up-regulated expression was also observed when correlated with the other clinico-pathological parameters. This progressive overexpression of Ki-67 provides an evidence of a multi-step deregulation of proliferation in HNSCC patients in Pakistani population. For gene-to gene interaction, we observed a statistically significant negative correlation between Ki-67 and SIRT3, Ki-67 and SIRT4 and Ki-67 and OGG1-2a gene. In current study, Ki-67 up-regulation has been linked with imbalances in mitochondrial tumor suppressor gene and malfunctioning of mitochondrial DNA repairing genes such as OGG1-2a, leading to an excessive proliferation in HNSCC cases which can result in more aggressive tumors and consequently the malignancy. In final part of present study, we observed a strong association between SIRT3, SIRT4, MTUS1 and OGG1-2a gene expression and lymph node metastasis. Essential characteristic of cancer is the ability to invade surrounding tissues and metastasize to regional lymph nodes and distant sites [32]. Detection of this local lymph node metastasis may thus prove to be pivotal for choosing appropriate treatment in individuals diagnosed with HNSCC.

In conclusion, our study demonstrated that down-regulation of selected mitochondrial TS genes such as SIRT3, SIRT4 and MTUS1 in HNSCC indicate aggressive tumor behaviors and may predict an unfavorable clinical outcome. Moreover, our findings also reveal a novel role of mitochondrial TS genes in head and neck carcinogenesis as a modulator of tissues oxidative stress and cell proliferation, supported by determining the levels of OGG1-2a gene and proliferation marker, Ki-67 in HNSCC cases respectively. This implicates mitochondrial TS genes as new potential therapeutic targets for treating HNSCC.

## Supporting Information

S1 Fig Hematoxylin and Eosin stained tissue sections and staining of regions showing the normal (A) and malignant (B) tissue of squamous cell carcinoma of head and neck. (TIF)

S2 Fig Melt profile of qPCR based analysis of (A) SIRT3, (B) SIRT4, (C) OGG1-2a, (D) MTUS1 and (E) Ki-67 in HNSCC. (TIF)

S1 Table List of primers specific gene SIRT3, SIRT4, OGG1-2a, MTUS1 and Ki-67 used in RT-PCR and qPCR. (DOCX)
