# Supplementary figures and images for: Loss of Mitochondrial Tumor Suppressor Genes Expression Is Associated with Unfavorable Clinical Outcome in Head and Neck Squamous Cell Carcinoma: Data from Retrospective Study
Source: PLoS One. 2016 Jan 19;11(1):e0146948. doi: 10.1371/journal.pone.0146948 (PMC4718451; doi:10.1371/journal.pone.0146948)

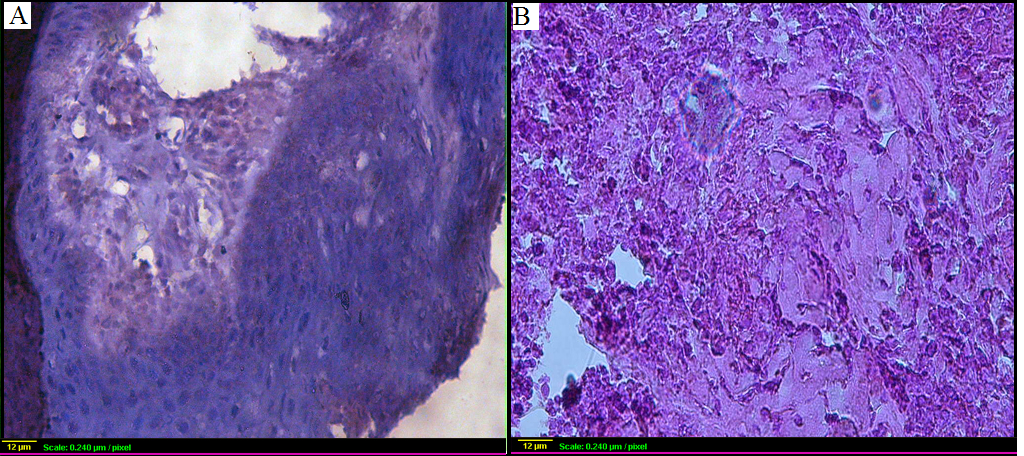

Supplement: S1 Fig — (TIF) [file pone.0146948.s001.tif]

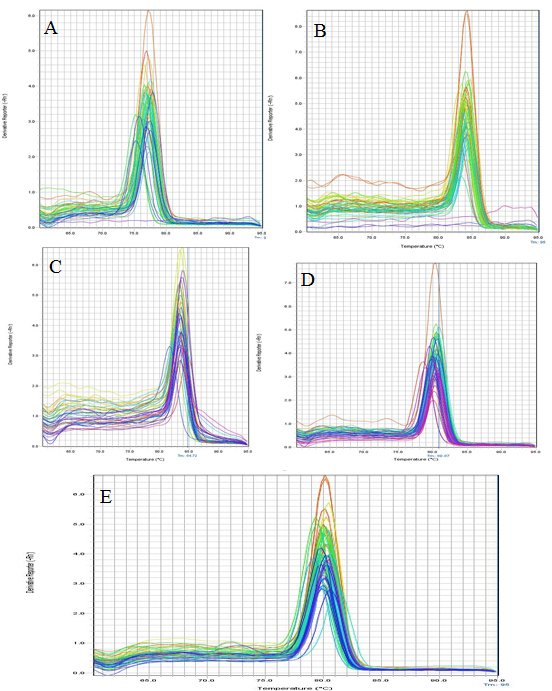

Supplement: S2 Fig — (TIF) [file pone.0146948.s002.tif]
