# Supplementary material for: Loss of Mitochondrial Tumor Suppressor Genes Expression Is Associated with Unfavorable Clinical Outcome in Head and Neck Squamous Cell Carcinoma: Data from Retrospective Study
Source: PLoS One. 2016 Jan 19;11(1):e0146948. doi: 10.1371/journal.pone.0146948 (PMC4718451; doi:10.1371/journal.pone.0146948)
Supplement: S1 Table — (DOCX) [file pone.0146948.s003.docx]

| **Gene** | **Primer Forward** | **Primer Reverse** | **Annealing Temperature** |
| --- | --- | --- | --- |
| SIRT3 | AAGGTGGAAGAAGGTCCATATC | GAATCAGCTCAGCTACATCCT | 60ºC |
| SIRT4 | CCCTGAGAAGGTCAAAGAGTTAC | TCTGACCTGTAGTCTGGTATCC | 55ºC |
| *Ki-67* | GCTGAGAACTCCTAAGGGAAAG | GCTGTGAAGCTCTGTAGGATAC | 58ºC |
| MTUS1 | GTGCTGAACCCAGAGCATAA | GGCGGGCTACTTAGAATCAAT | 60ºC |
| OGG1-2a | GACTGCATCTGCCTGATG | GTGC TAGTAAGCTGGCTTG | 60ºC |
| *β-actin* | TTCTCTGACCTGAGTCTCCTT | ACACCCACAACACTGTCTTAG | 55 ºC |

**S1 Table:** List of primers specific gene SIRT3, SIRT4, OGG1-2a, MTUS1 and Ki-67 used in RT-PCR and qPCR.
